# An overview of governance of noncommunicable diseases (NCDs) in West African countries

**DOI:** 10.11604/pamj.2023.44.203.36175

**Published:** 2023-04-26

**Authors:** Moussa Ouedraogo, Dia Sanou, Ousmane Ouedraogo, Urbain Zongo, Fatoumata Hama-Ba, Aly Savadogo

**Affiliations:** 1 Doctoral School of Sciences and Technology, Laboratory of Applied Biochemistry and Immunology (LABIA), University Joseph Ki-ZERBO, Ouagadougou, Burkina Faso,; 2 Nutrition Directorate, Ministry of Health and Public Hygiene, Ouagadougou, Burkina Faso,; 3 Food and Agriculture Organization (FAO), Subregional office, Eastern Africa, Addis Ababa, Ethiopia,; 4 United Nations Children's Fund (UNICEF), Ouagadougou, Burkina Faso,; 5 Food Technology Department, Institute for Research in Applied Sciences and Technologies, National Center for Scientific and Technological Research, Ouagadougou, Burkina Faso

**Keywords:** Governance, noncommunicable diseases (NCDs), ECOWAS, Burkina Faso

## Abstract

Noncommunicable diseases (NCDs) account for over 50% of total premature mortality in most low- and middle-income countries (LMICs). However, responses to the fight against NCDs are yet to be efficient in most of these countries. There is little published data on how this response is structured from a governance perspective in the context of global health systems. This study explored from existing research, the state of the governance in the fight against NCDs in the ECOWAS region. It consists of a review of articles published in peer-reviewed journals between 2010 and 2020 on ECOWAS countries. Of three hundred thirty-three (333) articles initially identified, eight (8) publications were included in these studies. There is a serious lack of information on the governance of NCDs in French-speaking countries such as Burkina Faso where no article has been identified. Of the 8 studies meeting the inclusion criteria, none has addressed the coherence of policies and programs. Seven (7) publications provided information on the component national NCDs policies, strategies and action plans, four (4) studies on the component of actors, interventions and the multisectoral coordination mechanism, five (5) on the issue of budget allocations and financing of NCD prevention and control interventions. Political commitment and government leadership has been discussed in three (3) publications. While some studies have provided information on the components of governance, it is important to remember that most of the studies were literature reviews and not empirical studies, which does not allow a better understanding of the situation of governance in each country. Designing an empirical study to answer some questions related to the governance of NCDs in the selected countries is necessary.

## Introduction

While infectious diseases and undernutrition problems for vulnerable people, especially children under 5, remain a major public health concern in poor countries, the data accumulated at the international level indicate an increase in mortality associated with non-communicable diseases linked to food in most developing countries. These countries seem to be experiencing an epidemiological and nutritional transition, similar to that of the industrialized countries in previous centuries [1]. Diet-related non-communicable diseases (NCDs) are today a major public health problem and a global development challenge [2]. Also known as chronic diseases associated with unhealthy diets, they include obesity, cardiovascular disease, diabetes, cancer, high blood pressure, and oral diseases [1]. According to experts of the Global Panel on Agriculture and Food Systems for Nutrition, the health risks of unhealthy diseases are greater than the combined risks of tobacco, alcohol, and unprotected sex [3]. Noncommunicable diseases account for over 50% of total premature mortality (i.e. deaths under age 60) in most low and middle-income countries (LMICs) [4,5]. There is little published data on how the response to NCDs is structured from a governance perspective in the context of global health systems [6]. Yet, the quality of governance in this area is critical to the success of the NCD response. Several concerns have been raised in the governance of NCDs in LMICs in the Western Pacific region. These include changes in NCD-specific institutional structures, unclear distribution of roles and responsibilities between NCD-specific structures and sectoral structures, insufficient alignment between NCD-specific plans and those of the different sectors, the lack of priorities, costs, and appropriate objectives, and finally, the effectiveness of multisectoral coordination mechanisms [6]. Although studies have been conducted in some African countries on NCDs, there remains a knowledge gap in terms of the current state of their governance in ECOWAS countries. This study proposes to carry out the inventory of research on governance in the fight against NCDs in the ECOWAS region order to inform future research.

## Methods

The methodological approach was based on the six key steps of a systematic review, namely: (i) formulate a review question and develop a search strategy based on explicit inclusion criteria for the identification of eligible studies; (ii) search for eligible studies using multiple databases and information sources, including gray literature sources, without any language restrictions; (iii) select studies, extract data and assess the risk of bias in a double manner using two independent reviewers to avoid random or systematic errors in the process; (iv) analyze the data using quantitative or qualitative methods (v) present the results in a summary of the results tables; (vi) interpret the results and draw conclusions [7]. This review was carried out from July to November 2021. The main research question was “What is the state of governance of NCDs in the ECOWAS countries?” A secondary question is “In which components of NCD governance there is the most knowledge gap?”

This systematic review focused on studies published in peer-reviewed journals between 2010 and 2020 on the governance of chronic diseases in the ECOWAS region. In this systematic review, the aim was to identify the most relevant articles published on policies and strategies, institutional arrangements, and coordination processes between the different actors and sectors in the management of non-communicable diseases. It concerned all the fifteen countries of the ECOWAS region. A systematic search of scientific articles published in English or French in peer-reviewed journals in three databases including PubMed, Google Scholar, and Web of Science was carried out. Table 1 shows the keywords and syntax that were used (in French, English, or Portuguese). The term “AND” has been applied in the search in order to capture only studies that deal with governance in relation to NCDs in target countries as defined in the conceptual framework of the study. The following inclusion criteria were considered for including articles in the review: 1) studies carried out or partially carried out in one or more countries in the ECOWAS region; 2) studies that include one or more governance indicators including: (i) political commitment and leadership, (ii) national policies, strategies, and action plan, (iii) multisectoral actors, interventions, and coordination mechanisms, (iv) common results framework or M&E system, (v) budget allocation and funding, and (vi) policy coherence; 3) studies published between 2010-2020. The initial literature search and title review as well as data extraction were performed by one author while the review phase of abstracts and full articles was independently performed by 2 authors and discussions led to the final list of items included in the analysis. The screening flowchart was produced with the PRISMA tool, which allows methodical mapping of the number of records identified, included, and excluded, and the reasons for the exclusions [8].

**Table 1:** the article search syntax

Politics and governance		Non-communicable diseases		ECOWAS countries		Period
Politics*, governance, coordinate* political commitment, leadership, leader* enabling environment, political economics, collaborate*, policy coherence, alignment; accountability; responsibility, incitation*; strategy*; action; intervention*; actors; funding, common framework, monitoring evaluation	**and**	Non-communicable diseases (NCD) chronic disease*, heart disease; overweight, obesity, diabetes, blood pressure, cancer, dyslipidemia	**and**	Benin, Burkina Faso, Cap vert, Ivory Coast, Gambia, Ghana, Guinea, Guinea Bissau, Liberia, Mali, Niger,Nigeria, Senegal, Sierra Leone, Togo, WAHO, ECOWAS UEMOA, CILSS	**and**	2010 - 2020

**Conceptual framework:** governance and leadership are two central elements of health systems [9]. The term governance is widely used in public administration, but the contours remain blurred with little agreement on the definition. The United Nations, for example, defines governance as “the exercise of economic, political, and administrative authority to manage a country´s affairs at all levels. It comprises mechanisms, processes, and institutions through which citizens and groups articulate their interests, exercise their legal rights, meet their obligations, and mediate their differences” [10]. In the field of health, its use is more recent and it targets three main functions, which are: i) the orientation of public action around the government's objective in health, ii) management with a view to optimizing the performance of the system, and iii) standardization to clarify relationships, foster cooperation and strengthen legitimacy. In short, it is a set of “systems, processes, and procedures put in place to guide the direction, management, and accountability of the health system” [11].

The concept of governance is inextricably linked to that of performance. It is about mobilizing knowledge, leadership, tools, resources, and levers of action so that the political and programmatic frameworks as well as the various stakeholders interact in synergy to orient public action as a whole, in the direction of continuous improvement of performance [12]. For this, governance is based on: (i) a management system (the set of rules that define the modalities of distribution of power and responsibilities); (ii) an information system (all the data and their operating system necessary for the organized action system to be intelligible and transparent at all times for professionals, managers, planners, patients, and population); and (iii) a financing system (all the incentives conveyed by the system's financing methods, the budget allocation mechanisms and the actors' payment mechanisms) [12,13].

**Figure 1:**
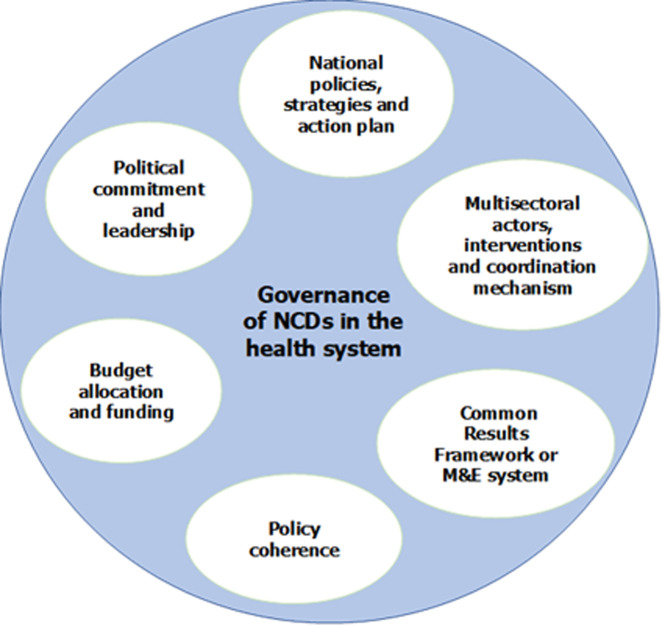
the conceptual framework for the analysis of noncommunicable disease governance

Most definitions incorporate institutional structures, relationships between public and private actors and/or organizations, decision-making processes, and motivations. Rani *et al*. (2022) proposed a conceptual framework for the analysis of the governance of NCDs in the health system around 3 major concepts, in particular: (i) national structures for supervision, planning, management, and monitoring, (ii) national policies, strategies, and action plans, and (iii) multisectoral coordination and partnerships [6]. Another conceptual framework for nutrition governance offers seven (7) concepts for analyzing nutrition governance. These are: (i) power, (ii) capacities, (iii) leadership, (iv) transparency of accountability, (v) knowledge and data, (vi) policy coherence, and (vii) political commitment [14]. For the purpose of this study and based on the country NCD context, we used a conceptual framework that builds on these two conceptual frameworks (Figure 1).

## Results

**Figure 2:**
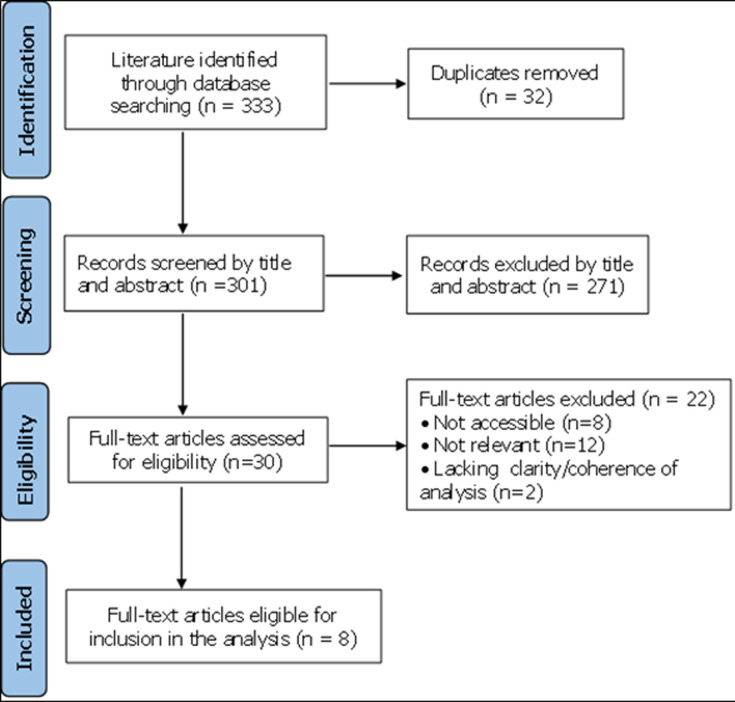
PRISMA flow diagram

The search in the various databases yielded 333 articles published in peer-reviewed journals. Due to the paucity of empirical studies on the subject, relevant review papers were considered. At the initial screening, 325 articles were excluded either because they did not include relevant governance of NCD and either because the research was not conducted in an ECOWAS country. After reviewing the articles against the previously defined inclusion criteria, eight (8) articles were included in this systematic review (Figure 2).

**Figure 3:**
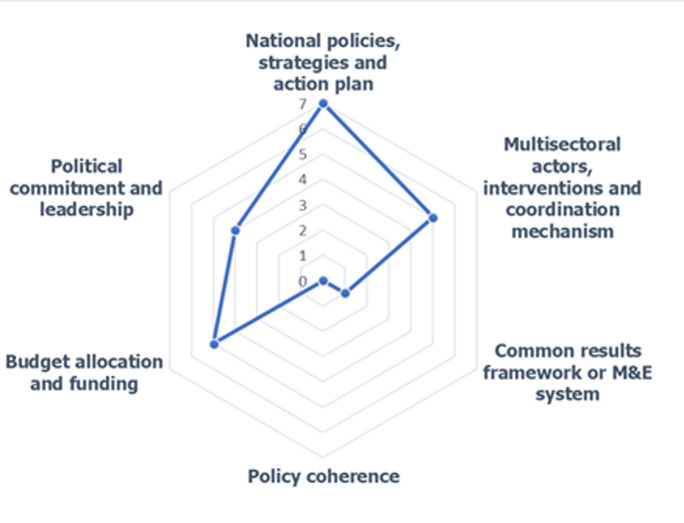
the distribution of publications obtained by component

***Characteristics of selected publications:*** of the 08 publications selected, four (4) were review papers and four (4) were empirical studies based on a mixed data collection approach (qualitative and quantitative). The review articles were conducted in Ghana [15] and Nigeria [16-18]. The four (4) empirical studies were carried out with a mixed data collection approach (qualitative and quantitative) and the data were collected both in ECOWAS countries (Cameroon, Nigeria, Togo, Guinea Bissau) also in countries outside ECOWAS such as Kenya and South Africa) [19-22]. Eleven (11) out of fifteen (15) ECOWAS countries i.e. 73% of ECOWAS countries including Burkina Faso, Mali, Benin, Cape Verde, Côte d'Ivoire, Gambia, Liberia, Niger, Senegal, Sierra Leone, Guinea have not been included in any study of the eligible articles published from 2010 to 2020. Regarding the different components of NCD governance as defined in this study, seven (7) publications addressed the political component, strategies, and action plans, five (5) explored the actors, interventions, and coordination component, one (1) included Common Results Framework or M&E System component, five (5) addressed budget allocations and four (4) examined political commitment and leadership. No publication exploring policy coherence was found. Figure 3 shows the distribution of publications obtained by the governance component as defined in this present study.

***Components of governance:*** the summary of articles by governance component is shown in Table 2, Table 2 (suite), Table 2 (suite 1).

**Table 2:** the matrix of key noncommunicable diseases (NCDs) governance components

No	Authors	National policies, strategies and action plan	Multisectoral actors, interventions and coordination mechanism	Common results framework or M&E system	Budget allocation and funding	Political commitment and leadership
1	Ghana	Developing an NCD policy, plan and strategy in Ghana; Identification of the fight against NCDs as priority by the MoH; establishment of a National NCD Technical Advisory Council and expert technical sub-committees on different NCDs; ratification of the framework convention for tobacco control (CCLAT) in 2004; completed the DHS which provided nationwide data on obesity in children and adult women and smoking in men; realization of the GDHS which provided information on the consumption of alcohol, fruits and vegetables in 2008	No coordinating body to put pressure for the implementation of NCDCP recommendations	Insufficiency in the monitoring and evaluation system for implementation; insufficiency in the process of national assessments of health services in Ghanaian health facilities	Considerable insufficiency of resources allocated to the fight against NCDs	NA
2	Ghana	Lack of a common business strategy to address NCDs due to the complexity of managing and preventing these diseases	Integrated approach to public-private partnerships in the fight against NCDs in Ghana; existence of a coalition of NGOs for health (CNGOH) working in tandem with state actors and other parastatal institutions; insufficient clear partnership with private organizations in a collaborative approach (multisectoral and multicultural)	NA	NA	NA

**Table 2(suite):** the matrix of key noncommunicable diseases (NCDs) governance components

No	Authors	National policies, strategies and action plan	Multisectoral actors, interventions and coordination mechanism	Common results framework or M&E system	Budget allocation and funding	Political commitment and leadership
3	Guinée Bissau	Insufficient validated epidemiological data on NCD prevalence to inform policy development	NA	NA	Insufficient of financial resources for NCD financial resources	NA
4	Nigeria	Development of national policies and plans for the prevention and control of NCDs; insufficient implementation of policies and action plans to end the increasing trend and burden of diabetes through effective primary care, especially in rural communities in Nigeria	Existence of civil society coalitions such as the Diabetes Association of Nigeria; the Nigerian NCD; Alliance and the Association of Medical Women of Nigeria	NA	NA	NA
5	Cameroon, Nigeria	Existence of anti-tobacco legislation; existence of a strategy to combat NCDs; insufficient data on NCD prevalence and risk factors	Creation of an advisory committee to coordinate the implementation of the 2015 tobacco law in 2016; Public sectors, private sector, associations, international organizations, local NGOs, research institutes and universities involved in the fight against NCDs; existence of NCD coordination departments/units within the ministries of health to lead policy development by engaging other sectors; insufficient awareness of the different sectors on their potential contribution for multisectoral action with complexity of coordination; lack of stronger coordination mechanisms with clear guidelines for engaging sectors for effective multisectoral action in NCD prevention	NA	Insufficient budget allocation for NCD prevention and control	Low political commitment

**Table 2(suite 1):** the matrix of key noncommunicable diseases (NCDs) governance components

No	Authors	National policies, strategies and action plan	Multisectoral actors, interventions and coordination mechanism	Common results framework or M&E system	Budget allocation and funding	Political commitment and leadership
6	Cameroon, Nigeria	Policy development in a consultative process with various stakeholders (other sectors than health); Insuffisance dans l'engagement des parties prenantes (difficile de déterminer la contribution réelle de chaque acteur organisationnel dans le processus); development of a tobacco policy	Existence of champions and advocates for the fight against NCDs influenced the political process; NGOs and civil society organizations have carried out important advocacy	NA	Insufficient technical resources and capacities	Strong leadership from the Ministry of Health (NCD prevention and control policy development process led by MoH); insufficient political commitment to support the development of policies for the prevention and control of NCDs
7	Nigeria	NA	NA	NA	NA	Insufficient commitment from government and non-governmental organizations to health care; Insufficient awareness among Nigerians on NCDs in general and diabetes in particular
8	Togo	Ratification of the WHO Framework Convention on Tobacco Control in 2005; Adoption of tobacco control legislation in 2010;	Insufficient collaboration with other sectors inside and outside government; Weak models of coordination of interaction between health and other sectors limited to information sharing	Lack of a common results framework leading to different expectations from stakeholders	Inadequate funding and overdependence on donors	Existence of political will

***National policies, strategies and action plans:*** seven (7) publications addressed national NCDs policies, strategies, and action plans, [16,17,19-23]. (W. Bosu, 2012) focused on national policy and program response to NCDs during the period 1992-2009 in Ghana. The study found that a number of policy and program initiatives to address NCDs have been put in place [23]. For example, Ghana has had an NCD Control and Prevention Program (NCDCP) since 1993. Ghana's VISION 2020 development program has included NCD issues as a priority. In addition to these reference documents, the author also mentioned the development of draft strategic frameworks for the prevention and control of NCDs from 2006 (documents being finalized) and the preparation of a draft national policy framework for NCDs in 2002 with technical support from WHO. The author also mentions the need to support programming and planning with scientific evidence, hence the conduct of surveys on NCD risk factors at the national level. Although many initiatives are underway in Ghana to tackle NCDs, another author noted in his publication the lack of a common business strategy to tackle NCDs due to the complexity of managing and preventing these diseases [16].

The existence of policies, strategies, and plans of action is also mentioned by other authors in other countries, in particular by Chineye and Ogu (2015) who reported in Nigeria, the development of national policies and plans for the prevention and control of NCDs even though they note with regret inadequate implementation of policies and action plans to end the growing burden of NCDs, especially in rural communities in Nigeria [17]. The same is true for Juma and colleagues who note in Kenya, South Africa, Cameroon, Nigeria, and Malawi, the existence of legislation against tobacco, an NCD control strategy as well as the creation of an advisory committee to coordinate the implementation of the 2015 Tobacco Act [20,21]. However, the authors stress that stakeholder commitments have not been well documented, so it is difficult to determine the real contribution of each organizational actor in the process. In Togo, Sanni mentions the ratification of the WHO framework convention for tobacco control in 2005, and the adoption of legislation on tobacco control in 2010, but does not mention a policy, strategy, or action plan in place for NCD control [22]. Correira, on the other hand, regretted the lack of validated epidemiological data on the prevalence and risk factors of NCDs to inform policymaking in Guinea-Bissau [19].

***Actors, interventions and multisectoral coordination mechanism:*** four (4) studies have addressed this component of governance which relates to actors, interventions, and the multisectoral coordination mechanism [16,20-23]. These studies mainly addressed the actors and the multisectoral coordination mechanism. The question of the interventions implemented did not emerge. In Ghana, Bosu mentioned the disinterest of many development partners in NCD issues, moreover, the author underlines the absence of a coordinating body for the implementation of the recommendations of the program for the control and prevention of NCDs [15]. Adjéi, on the other hand, notes the existence of a coalition of NGOs for health (CNGOH) working in tandem with state actors and other parastatal institutions in the fight against NCDs in Ghana. However, he underlined a lack of clear partnerships with private organizations in a collaborative approach (multisectoral and multicultural) [16]. Juma *et al*. mention some important players in the fight against NCDs in Kenya, South Africa, Cameroon, Nigeria, and Malawi. These include the public sector, the private sector, associations, international organizations, local NGOs, research institutes and universities, resource persons designated as NCDs champions, and advocates. Bosu also clarified the existence of NCD coordination departments/units within the ministries of health to lead policy development by engaging all sectors. However, he noted a lack of awareness of the different sectors on their potential contribution to multisectoral action with a complexity of coordination. The authors also highlighted an insufficiency of the coordination mechanism characterized by an absence of clear guidelines for the engagement of sectors with a view to multisectoral action [20,21]. In Togo, Sanni did not identify the actors involved in the implementation of NCD interventions. However, the author highlighted shortcomings in collaboration with other sectors within and outside government. They also note a limited interaction in information sharing between the health sector and other sectors as well as different expectations of stakeholders [22]. The involvement of sectors other than health in addressing NCDs in countries does not seem very clear based on these articles.

***Common results framework or monitoring, evaluation, and accountability system:*** Bosu´s publication in Ghana addressed the issue of monitoring and evaluation. The author noted an insufficiency in the monitoring and evaluation mechanism for implementation. For example, he mentioned the exclusion of NCDs in recent national evaluations of healthcare services at health facilities in Ghana and pointed out that no formal evaluation of the NCDCP program has been carried out since its inception in 1992 (compared to the national tuberculosis control program created in 1994 which has been evaluated at least 5 times) [23].

***Budget allocation and funding:*** five (5) studies have addressed the issue of budget allocations and financing of NCD prevention and control interventions [19-23]. Bosu in Ghana addressed the issue of budget allocation and funding for the fight against NCDs. Although the author did not mention the amount of money allocated to NCD control. The author noted a considerable shortfall in resources allocated to NCD control in Ghana compared to that allocated to TB control, for example (W. Bosu, 2012). The same goes for the publications of Coreira and Juma [19-21]. Sanni mentioned the heavy dependency on donors in the fight against NCDs [22].

***Political commitment and leadership:*** political commitment and government leadership have been discussed in three (3) publications [18,20,21]. Juma noted strong leadership from the Ministry of Health (NCD Prevention and Control Policy Development Process led by MoH). However, he mentioned a lack of commitment and political will for the prevention and control of NCDs. In Nigeria, Olufemi also noted insufficient engagement of government and non-governmental organizations and insufficient awareness among Nigerians on NCDs in general and diabetes in particular.

## Discussion

This review shows that there are huge publication gaps related to the governance of NCD in ECOWAS region, while their prevalence of increasing. Only 8 articles were found relevant to NCD governance in the period 2010-2020 in the 15 ECOWAS countries with a high number of articles being review papers (4/8). Six (6) of the 8 publications were produced in English-speaking countries, in particular Ghana and Nigeria. Only one study considered in addition to an English-speaking country (South Africa), a French-speaking country Togo and one study considered a Portuguese-speaking country Guinea Bissau. The “National policies, strategies, and action plan” component was addressed in seven (7) publications followed by the “actors, interventions and multisectoral coordination mechanism” component and the “Budget allocation and financing” component examined respectively in five (5) publications. The “Political commitment and leadership” component and the “Common results framework or M&E system” component were discussed respectively in 4 and 1 publications.

Several ECOWAS countries have developed policies and programs to combat NCDs. They seem to have realized the value of rethinking policies at the national level to address the issue of NCDs. In 2017, for example, fifteen Member States of the African Region including Benin, Burkina Faso, Côte d'Ivoire, Ghana, and Niger, had integrated multisectoral national operational policies and plans to fight against NCDs [24]. This is the case, for example, of Ghana, whose history is marked by an extraordinary dynamic in the development of initiatives in favor of the fight against NCDs but whose insufficient institutional framework and integration of multisectoral actors and multicultural makes implementation of international provisions inoperative [15,23]. Burkina Faso has also developed an integrated plan to fight against NCDs which covers the period 2016-2020. Neither the process of developing the plan, the mid-term evaluation, the coherence, nor the actors involved have been the subject of research to inform decision-makers about the gaps in terms of compliance with international commitments. For the most part, these policies have been developed without really being evidence-based since data on the prevalence of NCDs is sorely lacking in some countries [19,25]. This does not guarantee optimal decision-making in the fight against NCDs.

Many authors call for a diversification of actors with an effective coordination mechanism for the implementation of interventions [16,20]. This is fully justified since, according to WHO, without an effective coordination mechanism, it would be difficult to achieve the SDGs in terms of prevention and control of NCDs [26]. From this review, it unclear what is the level of involvement of non-health related sectors and what contribution they are making to the NCDs. However, several questions remain unanswered in the work of these authors, among others who are the key players in the process? What are the sectors involved and what are the roles and responsibilities of each actor? What coordination mechanism for an effective response to the NCD in a context or in most of the ECOWAS countries, the fight against malnutrition remains the priority? How to obtain and maintain their commitment and their involvement in the response?

Multisectoral action requires the development of a common results framework around various actors' contributions to improving NCD indicators [27]. However, the multisectoral response to non-communicable diseases in different countries is insufficient. The prevention and control of these non-communicable diseases largely remain the prerogative of the health sector, with little to no involvement of the other sectors in this effort [24]. No author seems to address this issue. We have had no information on the process of drafting this consensus document, let alone the challenges of its implementation. The same is true for policy coherence which has not been addressed in any of the articles included in this review. It is true that several ECOWAS countries have developed policies, plans and programs for the prevention and control of NCDs but the results are mixed and the prevalence continues to increase [15,23]. There is however no clear information on how these policies and programs are aligned and synergetic in a multisectoral and multistakeholder environment. The lack of coherence of policies can affect the redistribution of resources within political subsystems, creating competition between interest groups, a competition which often will not serve, neither the interest groups nor the government [28].

All of the authors who have addressed the issue of budget allocations have recognized that the resources allocated to the fight against NCDs are insufficient. The Global Health Expenditure Report also makes the same observation [29]. Unfortunately, the author's work did not specify the amount allocated to the fight against NCDs, the sectors and technical and financial partners involved, nor the financial impact on household income. The issue of innovative financing mechanisms such as the taxation of beverages, and tobacco products was also not discussed. No policy or program is as coherent as it can achieve its objectives without a substantive budgetary allocation either from the government or its development partners, hence the need to examine in detail this component through empirical research in order to identify the funding gaps. Strong political commitment and government leadership are one of the conditions for the good governance of NCDs [26]. The researchers mostly noted weak political will and recognized that more political leadership and commitment and partners would be needed to catalyze and maintain the focus on NCD [20,21,30]. However, like other components of governance, the researchers did not examine the challenges and bottlenecks of strong commitment and leadership in the fight against NCDs with an analytical tool, political commitment like the one proposed by [31].

Although all ECOWAS countries have subscribed to the international provisions which call on the Member States of the United Nations to improve the governance of NCDs in all its components (WHO, 2013), the majority of Member States, have achieved little. progress or have made no progress at all [24]. Most researchers have set their sights on the problem of undernutrition and its many consequences. Yet with the rapid socio-economic transitions occurring in sub-Saharan Africa, there is a risk that the increasing prevalence of NCDs will overwhelm the already struggling health services and have adverse consequences for individuals and economies [1,32]. However, this study has some limitations that should be highlighted. The methodological approach to retrieving articles is based on their availability online in reliable databases, which de facto exclude articles not published or published in local journals which are not well-known peer-reviewed journals. Indeed, in Africa, important work is done and not published in international journals, limiting their access. Many researchers also published in local and regional journals which are not available in most search engines for peer-reviewed journals. The other limit is the inclusion of articles reviewed in the analysis, which did not provide all the information to better understand the component(s) examined. For example, some publications address the insufficiency of resources allocated to the management of NCDs, however, neither the amount allocated the gaps in financing needs, nor the attention of donors for NCDs are examined. This creates a lack of information to better understand the environment of each component.

## Conclusion

This review suggests that very few peer-reviewed articles have looked at the governance of NCDs in the ECOWAS region. This suggests that there are many unanswered questions that could be the subject of research questions. English-speaking ECOWAS countries have produced more data on the governance of NCDs than French-speaking countries where there is a serious lack of information on all components of governance. Most of the studies reviewed provided information on the components: (i) political commitment and leadership, (ii) national policies, strategies, and action plan, (iii) multisectoral actors, interventions, and coordination mechanisms, and (iv) budget allocation and funding even if this information remains insufficient to better understand the situation in each country. The components: (i) common results Framework or M&E system, and (ii) policy coherence have not received much attention from researchers. This could be a considerable limit to the implementation of policies and strategic plans when we know the role of policy coherence and the M&E system in achieving results. Researchers should take note of these in order to provide answers through empirical studies to fuel discussions for more effective governance of NCDs.

### What is known about this topic

- *There is little published data on how response to NCDs is structured from a governance perspective in the context of global health systems in low-income countries;*
- *Yet, the quality of governance in this area is critical to the success of the NCD response;*
- *The shortcomings mainly concern institutional structures, unclear distribution of roles and responsibilities between NCD-specific structures and sectoral structures, insufficient alignment between NCD-specific plans and those of the different sectors, the lack of priorities, costs and appropriate objectives and finally, the effectiveness of multisectoral coordination mechanisms*.

### What this study adds

- *Few researchers have looked into the issue of NCD governance in the ECOWAS region;*
- *Few published data on how the response to NCDs is structured from a governance perspective in the context of national health systems;*
- *The research gaps on the governance of NCDs identified will feed the discussions during the next revisions of the plans to fight against NCDs, in particular in Burkina Faso where the plan has come to an end*.
